# Precise measurement of gene expression changes in mouse brain areas denervated by injury

**DOI:** 10.1038/s41598-022-26228-5

**Published:** 2022-12-29

**Authors:** Jessica Schlaudraff, Mandy H. Paul, Thomas Deller, Domenico Del Turco

**Affiliations:** Institute of Clinical Neuroanatomy, Neuroscience Center, Goethe-University Frankfurt, Theodor-Stern-Kai 7, 60590 Frankfurt, Germany

**Keywords:** Neuroscience, Molecular neuroscience, Regeneration and repair in the nervous system

## Abstract

Quantitative PCR (qPCR) is a widely used method to study gene expression changes following brain injury. The accuracy of this method depends on the tissue harvested, the time course analyzed and, in particular on the choice of appropriate internal controls, i.e., reference genes (RGs). In the present study we have developed and validated an algorithm for the accurate normalization of qPCR data using laser microdissected tissue from the mouse dentate gyrus after entorhinal denervation at 0, 1, 3, 7, 14 and 28 days postlesion. The expression stabilities of ten candidate RGs were evaluated in the denervated granule cell layer (gcl) and outer molecular layer (oml) of the dentate gyrus. Advanced software algorithms demonstrated differences in stability for single RGs in the two layers at several time points postlesion. In comparison, a normalization index of several stable RGs covered the entire post-lesional time course and showed high stability. Using these RGs, we validated our findings and quantified glial fibrillary acidic protein (*Gfap*) mRNA and allograft inflammatory factor 1 (*Aif1/Iba1*) mRNA in the denervated oml. We compared the use of single RGs for normalization with the normalization index and found that single RGs yield variable results. In contrast, the normalization index gave stable results. In sum, our study shows that qPCR can yield precise, reliable, and reproducible datasets even under such complex conditions as brain injury or denervation, provided appropriate RGs for the model are used. The algorithm reported here can easily be adapted and transferred to any other brain injury model.

## Introduction

Quantitative polymerase chain reaction (qPCR) has emerged as a standard for precise analysis of quantitative changes in gene expression, especially when only a few target genes are examined or when a small amount of tissue is used^1–4^. To ensure accurate and reproducible data, qPCR experiments comprise several aspects, including experimental design, sample preparation and data analysis^5,6^. The use of reference genes (RGs) is the most common strategy to normalize target gene expression of interest. However, their suitability must be experimentally validated^7–11^. Several studies demonstrated that expression levels of some commonly used RGs might vary considerably depending on the specific condition investigated, e.g., traumatic brain injury^12–16^. Unfortunately, there is no ideal RG for all experimental conditions and stability of RGs cannot be simply assumed. Under conditions of brain injury, the situation may be even more complex, because gene expression for multiple pathways of injury and repair vary with time following lesion^17–19^ as well as with age^15,20–22^. Furthermore, gene expression levels of neurons and glial cells depend on their distance from the lesion^23^ and, thus, studies using laser microdissected tissues may provide more precise, robust and reproducible results than studies using homogenized larger tissue blocks^15,24^.

To demonstrate the usefulness of this combined approach in a proof-of-principle experiment, we investigated the expression stability of candidate RGs in the dentate gyrus (DG) following entorhinal cortex lesion, a classical model for neural reorganization of the brain after injury^20,25–28^. In this model, transection of the perforant path results in the denervation of the outer molecular layer (oml) of the DG, accompanied by a strong glial reaction^29,30^. Candidate RGs were chosen based on earlier entorhinal lesion studies of the rodent brain^16,31,32^ and because of other relevant brain injury studies^12–14,33–36^. In contrast to previous studies using total hippocampal tissue following entorhinal lesion^32^, we used laser microdissection to harvest two hippocampal layers, i.e., the granule cell layers, where the denervated granule cells are located and the oml, i.e., the zone of denervation where strong glial reactions have been reported^29,37,38^. Expression stability of ten putative RGs, i.e., actin, beta (*Actb*), aminolevulinic acid synthase 1 (*Alas1*), beta-2 microglobulin (*B2m*), glyceraldehyd-3-phosphate dehydrogenase (*Gapdh*), hypoxanthine guanine phosphoribosyl transferase (*Hprt*), phosphoglycerate kinase I (*Pgk1*), peptidyl propyl isomerase A (*Ppia*), ribosomal protein L13A (*Rpl13a*), succinate dehydrogenase complex subunit A (*Sdha*) and transferrin receptor (*Tfrc*), were evaluated using commercially available software algorithms, i.e., NormFinder^39^ and geNorm^40^, and consensus ranking analysis^41^. To validate our findings, two prominent glial genes were chosen, i.e., glial fibrillary acidic protein (*Gfap*), a marker for reactive astrocytes, and allograft inflammatory factor 1 (*Aif1*)—better known as ionized calcium binding adaptor molecule 1 (*Iba1*)—a marker for activated microglia cells. Both genes are known to be upregulated after brain injuries and following entorhinal lesion^29,42–44^. Our study showed that single RGs were not sufficiently stable to study the time course of these genes over the entire postlesional time. In contrast, an index of several RGs yielded reliable, reproducible, and accurate results. This index should be used for normalization of qPCR data following entorhinal denervation and similar indexes should be identified using the algorithm described here for other lesion models to provide accurate qPCR data.

## Results

### Transection of the perforant path denervates the outer molecular layer of the dentate gyrus and induces strong and layer-specific astro- and microglial responses

**Figure 1:**
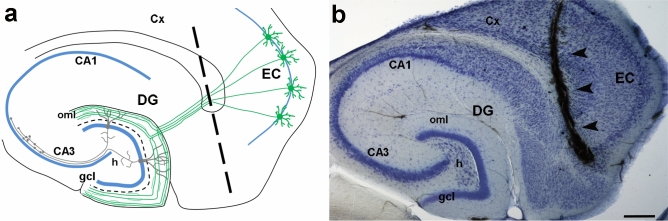
Entorhino-hippocampal denervation model. (**a**) Schematic of a horizontal brain section illustrating the entorhino-hippocampal denervation model. The perforant path (green) originates from stellate neurons in the entorhinal cortex (EC) and terminates on distal parts of granule cell dendrites in the outer molecular layer (oml) of the dentate gyrus (DG). The transection site of the perforant path is indicated by a dotted line. (**b**) Representative brain section (horizontal plane, cresyl violet staining) after transection of the perforant path as indicated in (**a**). Note the distance of the dentate gyrus from the lesion site (arrowheads). CA1, CA3: cornu ammonis subregions, gcl: granule cell layer, h: hilus; Cx: cortex. Scale bar: 250 μm.

**Figure 2:**
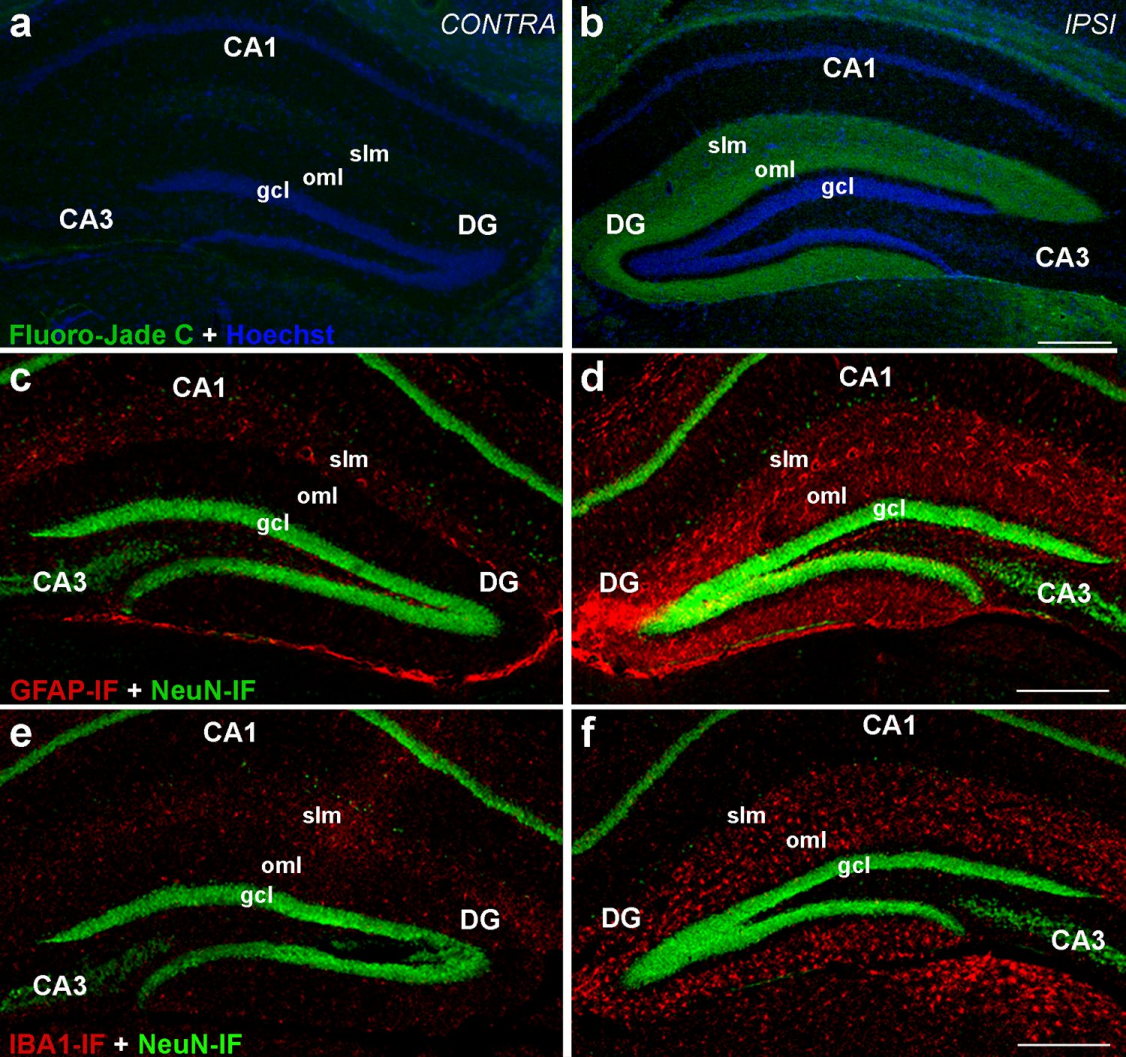
Entorhinal denervation leads to a layer-specific denervation and a layer-specific glial reaction in the outer molecular layer of the dentate gyrus. (**a**, **b**) Unilateral transection of the perforant path leads to layer-specific denervation in the dentate gyrus (DG) as shown by Fluoro-Jade C staining (green) at 7 days post lesion. The contralateral hippocampus is shown as control (**a**). Note that Fluoro-Jade C staining is seen in the outer molecular layer (oml) of the DG only in the ipsilateral hippocampus (**b**). Nuclei (blue) were counterstained with Hoechst 33342. (**c**–**f**) Layer-specific activation of glial cells in the DG oml following entorhinal lesion (7 days post lesion). Sections were immunolabeled against glial fibrillary acid protein (GFAP) (**c**, **d**) and ionized calcium-binding adapter molecule 1 (IBA1) (**e**, **f**), marker molecules for reactive astrocytes and activated microglia, respectively. The contralateral hippocampus is shown as control (GFAP (**c**); IBA1 (**e**)). Strong layer-specific activation of both glial cell types is seen in the denervated oml of the ipsilateral side (GFAP (**d**); IBA1 (**f**)). For counterstaining, NeuN (neuronal marker, green) was used. CA1, CA3: cornu ammonis subregions, slm: stratum lacunosum moleculare, gcl: granule cell layer. Scale bars: 250 μm.

The entorhino-hippocampal lesion model^26,29^ was used to examine the expression stability of putative RGs in the denervated adult mouse DG (Fig. 1). Transection of the perforant path leads to layer-specific denervation of the outer part of the dentate molecular layer, the oml (Fig. 1a). Quality and completeness of entorhinal denervation were demonstrated using Fluoro-Jade C staining (Fig. 2a,b). A strong glial reaction was observed in the DG ipsilateral to the lesion (ipsi) compared to the DG contralateral to the lesion (contra). Immunofluorescence labeling for GFAP, a classical marker for reactive astrocytes (Fig. 2c,d), as well as for IBA1, a widely used marker for activated microglia (Fig. 2e,f) revealed strongly increased reactivity for both markers in the denervated zone at 7 days postlesion.

### Quantitative expression analysis of putative reference genes in the laser microdissected granule cell layer and outer molecular layer of the dentate gyrus

**Figure 3:**
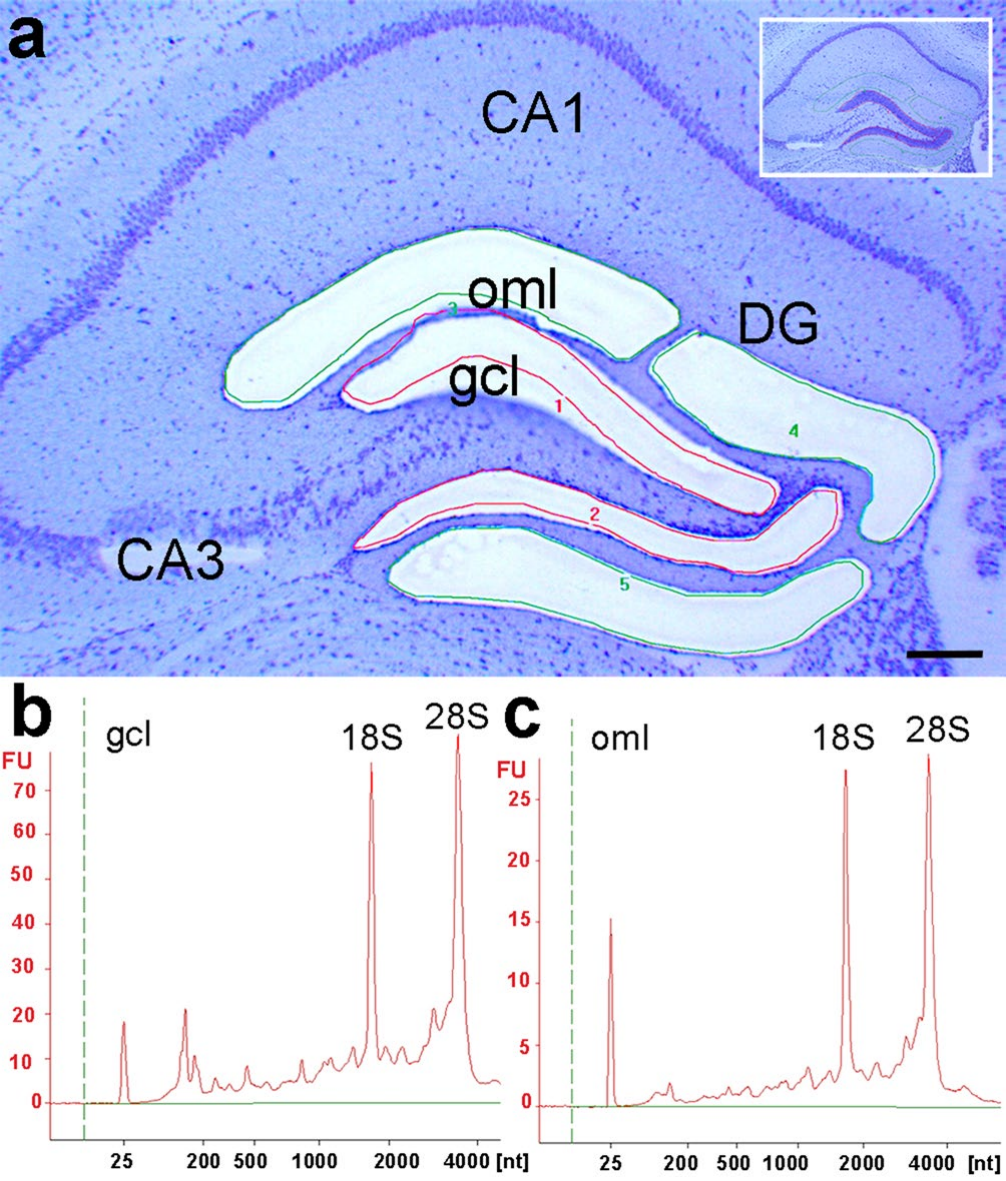
Laser microdissection of dentate subregions. (**a**) Hippocampal section (coronal plane, dorsal part of the hippocampus, cresyl violet staining) before (inset) and after laser microdissection of the granule cell layer (gcl) and the outer molecular layer (oml). (**b**, **c**) RNA integrity analysis of total RNA isolated from the dissected gcl and oml demonstrating highly intact RNA (RIN-values: 8.15–8.3) as determined using the Agilent 2100 Bioanalyzer system. DG: dentate gyrus, CA1, CA3: cornu ammonis subregions). Scale bar: 200 μm.

**Figure 4:**
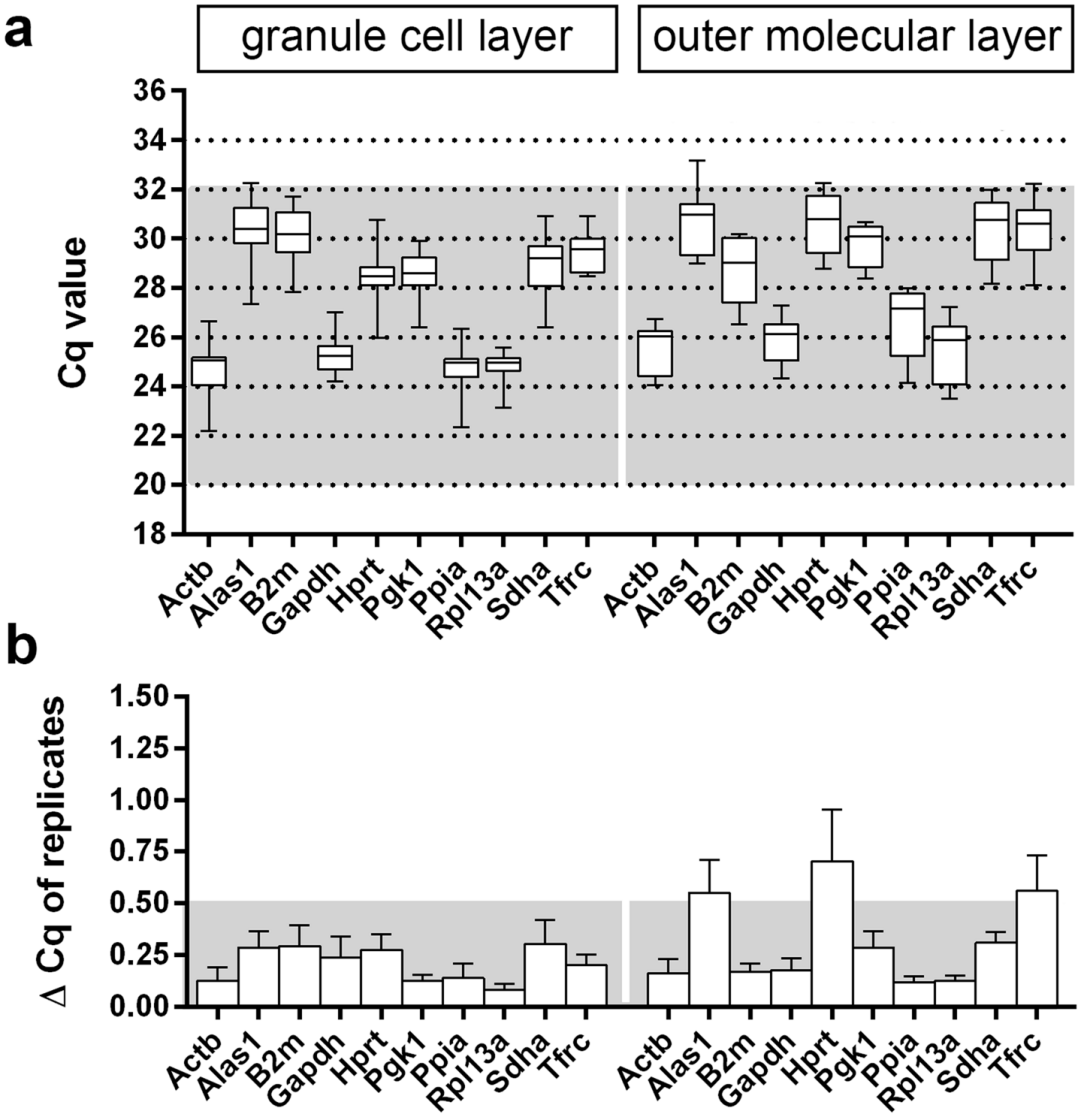
Cq values of candidate reference genes in microdissected dentate gyrus regions. (**a**) Box plots represent absolute Cq values of ten putative reference genes in laser-microdissected granule cell layers and outer molecular layers of the dentate gyrus. Optimal range of Cq values is highlighted in grey. (**b**) ∆ Cq values of replicates (doublets) of candidate reference genes. Optimal range of ΔCq values is highlighted in grey. Cq, quantification cycle. N = 6 animals per layer.

At first, expression levels of putative RGs were evaluated in the granule cell layer (gcl) and oml of the DG to prove for abundance as well as to examine the variability of selected RGs. Laser microdissection was used to dissect and harvest the two dentate layers (Fig. 3a). RNA integrity analysis confirmed the high quality of isolated RNA (RIN values > 8) from gcl and oml samples used for further qPCR experiments (Fig. 3b,c). Quantitative PCR amplification demonstrated absolute quantification cycle (Cq) values in the standard range from 20 to 32 for all putative RGs across the tissue samples (Fig. 4a). ΔCq values of replicates for the dentate gcl were below a cut-off of 0.5 and, thus, all RGs were included in our evaluation (Fig. 4b). In the dentate oml samples, however, three putative RGs, i.e., *Alas1*, *Hprt*, and *Tfrc,* had ΔCq values greater than 0.5 and, thus, these candidates had to be excluded (Fig. 4b).

### Expression stability of putative reference genes in the granule cell layer of the dentate gyrus after entorhinal denervation

**Figure 5:**
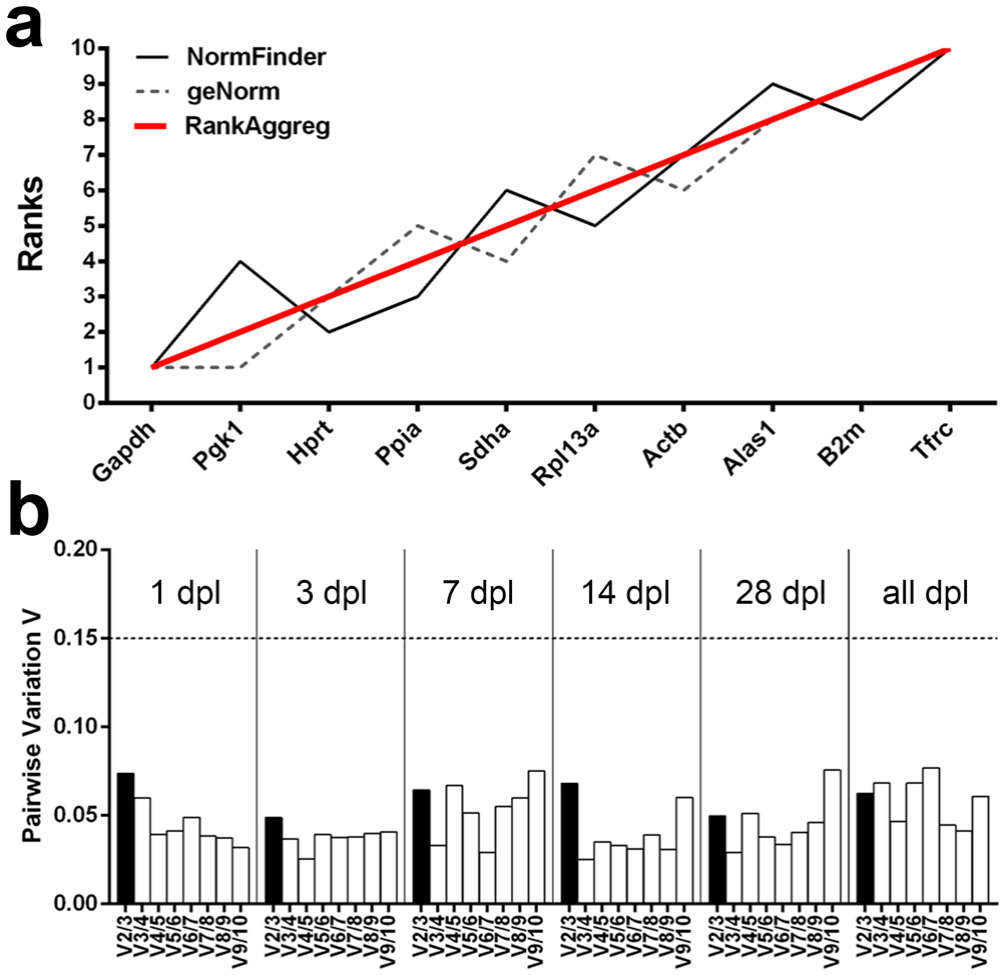
Evaluation of consensus ranking and determination of the optimal number of reference genes by pairwise variation for the granule cell layer after entorhinal denervation. (**a**) Expression stability of candidate reference genes for all time-points after entorhinal denervation analyzed by NormFinder and geNorm. RankAggreg was used for consensus ranking analysis. (**b**) Determination of the optimal number of reference genes recommended for accurate normalization based on the analysis of pairwise variation (V) implemented in geNorm. Every bar represents the change in normalization accuracy by stepwise inclusion of an additional reference gene according to the ranking at specific time points (1, 3, 7, 14, 28 days post lesion, dpl) or for the entire lesion period (all dpl). A cut-off of 0.15 (dashed line) was used below the inclusion of an additional reference gene was not required for proper normalization. Pairwise variation analysis revealed a minimum number of three reference genes needed for accurate normalization.

Ten putative RGs were tested for their expression stability in the dentate gcl at different time points following entorhinal denervation, i.e., 1, 3, 7, 14 and 28 days. RGs were ranked according to the stability values calculated by NormFinder and by averaged expression using geNorm. RG stability measurements calculated by these algorithms were then used for a consensus ranking analysis employing RankAggreg (Table 1). Furthermore, expression stability of candidate RGs was analyzed for the entire lesion period, i.e., from 1 to 28 dpl (Fig. 5a). Calculation of pairwise variation analysis based on the consensus ranking list for each time-point (see Table 1) and, in addition, for all time-points following lesion revealed a minimum number of at least three stable RGs required for normalization (Fig. 5b). NormFinder identified *Hprt* and *Ppia* as the best pair of RGs, whereas geNorm determined *Gapdh*, *Pgk1* and *Hprt* as the most stable RGs for qPCR normalization. Summarized by RankAggreg, the consensus stability list of RGs for the entire lesion period reads as follows (most stable to least stable): *Gapdh–Pgk1–Hprt–Ppia–Sdha–Rpl13a–Actb–Alas1–B2m–Tfrc.*

**Table 1:** Ranking of candidate reference genes according to their expression stability in the dentate granule cell layer following entorhinal denervation.

dpl	Ranking	NormFinder		geNorm		RankAggreg
		Stability value		M-value		Ranks
1 dpl	1	Gapdh*	0.056	***Gapdh* + *Pgk1***	0.166	***Gapdh***
	2	B2m*	0.068			***Pgk1***
	3	***Hprt***	0.072	***Rpl13a***	0.197	***B2m***
	4	***Pgk1***	0.076	***B2m***	0.217	***Hprt***
	5	***Rpl13a***	0.087	***Hprt***	0.242	***Rpl13a***
	6	***Alas1***	0.091	***Sdha***	0.255	***Sdha***
	7	***Sdha***	0.097	***Actb***	0.282	***Alas1***
	8	***Actb***	0.125	***Alas1***	0.306	***Actb***
	9	***Ppia***	0.134	***Ppia***	0.323	***Ppia***
	10	***Tfrc***	0.137	***Tfrc***	0.336	***Tfrc***
3 dpl	1	Sdha*	0.071	***Actb* + *Pgk1***	0.073	***Pgk1***
	2	***Hprt***	0.088			***Actb***
	3	Pgk1*	0.104	***Gapdh***	0.082	***Sdha***
	4	***Actb***	0.110	***Sdha***	0.121	***Hprt***
	5	***Gapdh***	0.116	***Hprt***	0.141	***Gapdh***
	6	***Ppia***	0.125	***Ppia***	0.178	***Ppia***
	7	***Rpl13a***	0.167	***Rpl13a***	0.210	***Rpl13a***
	8	***B2m***	0.180	***B2m***	0.241	***B2m***
	9	***Alas1***	0.210	***Alas1***	0.274	***Alas1***
	10	***Tfrc***	0.233	***Tfrc***	0.308	***Tfrc***
7 dpl	1	***Gapdh***	0.059	***Pgk1* + *Sdha***	0.129	***Pgk1***
	2	***Pgk1***	0.133			***Sdha***
	3	***Hprt***	0.133	***Hprt***	0.144	***Gapdh***
	4	Sdha*	0.165	***Gapdh***	0.171	***Hprt***
	5	***Ppia***	0.197	***Actb***	0.240	***Actb***
	6	Rpl13a*	0.199	***Rpl13a***	0.274	***Ppia***
	7	***Actb***	0.219	***Ppia***	0.286	***Rpl13a***
	8	***B2m***	0.348	***B2m***	0.353	***B2m***
	9	***Alas1***	0.376	***Alas1***	0.403	***Alas1***
	10	***Tfrc***	0.491	***Tfrc***	0.478	***Tfrc***
14 dpl	1	***Rpl13a***	0.073	***Gapdh* + *Pgk1***	0.065	***Gapdh***
	2	***Gapdh***	0.081			***Pgk1***
	3	***Pgk1***	0.109	***B2m***	0.117	***Rpl13a***
	4	***B2m***	0.130	***Actb***	0.144	***B2m***
	5	Hprt*	0.141	***Rpl13a***	0.168	***Actb***
	6	***Actb***	0.149	***Hprt***	0.186	***Hprt***
	7	Ppia*	0.167	***Sdha***	0.202	***Sdha***
	8	***Alas1***	0.168	***Alas1***	0.228	***Ppia***
	9	***Sdha***	0.181	***Ppia***	0.253	***Alas1***
	10	***Tfrc***	0.357	***Tfrc***	0.326	***Tfrc***
28 dpl	1	***Hprt***	0.086	***Actb* + *Pgk1***	0.108	***Hprt***
	2	Ppia*	0.095			***Actb***
	3	***Gapdh***	0.104	***Gapdh***	0.133	***Gapdh***
	4	Actb*	0.107	***Hprt***	0.149	***Pgk1***
	5	***B2m***	0.137	***Sdha***	0.174	***Ppia***
	6	***Pgk1***	0.160	***Ppia***	0.212	***B2m***
	7	***Rpl13a***	0.185	***B2m***	0.232	***Sdha***
	8	***Sdha***	0.187	***Rpl13a***	0.263	***Rpl13a***
	9	***Alas1***	0.313	***Alas1***	0.301	***Alas1***
	10	***Tfrc***	0.452	***Tfrc***	0.396	***Tfrc***

Ranking of candidate reference genes for the granule cell layer at specific time-points (1, 3-, 7-, 14- and 28-days post lesion (dpl)) after entorhinal denervation. Reference genes were ranked according to (1) stability values calculated by NormFinder, (2) averaged expression by geNorm and (3) using a consensus ranking analysis by RankAggreg. Asterisks (left column) indicate the RGs selected as ‘best combination of two RGs’ by NormFinder.

### Evaluation of reference gene expression stability in the outer molecular layer of the dentate gyrus after entorhinal denervation

**Figure 6:**
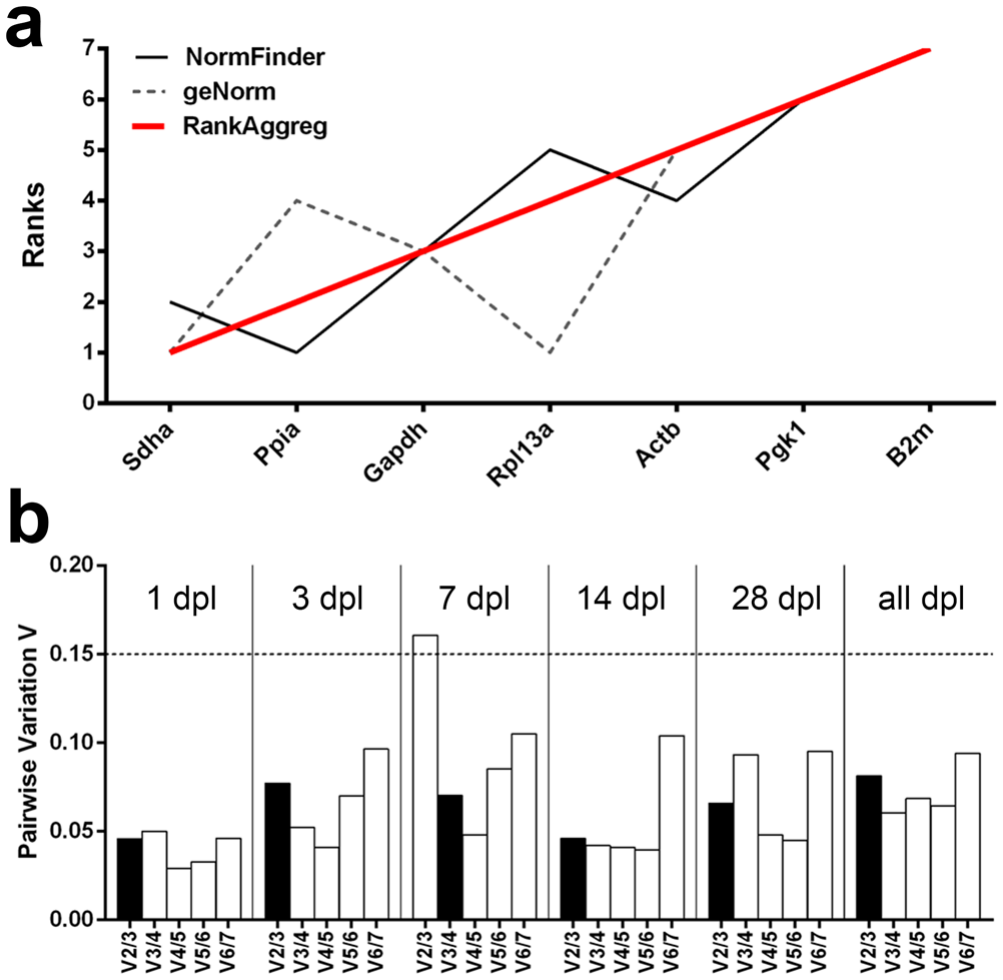
Evaluation of consensus ranking and determination of the optimal number of reference genes by pairwise variation for the outer molecular layer after entorhinal denervation. (**a**) Expression stability of candidate reference genes for all time-points after entorhinal denervation analyzed with NormFinder and geNorm. RankAggreg was used for consensus ranking analysis. (**b**) Determination of the optimal number of reference genes recommended for accurate normalization based on the analysis of pairwise variation (V) implemented in geNorm. Every bar represents the change in normalization accuracy by stepwise inclusion of an additional reference gene according to the ranking at specific time points (1, 3, 7, 14, 28 days post lesion, dpl) or for the entire lesion period (all dpl). A cut-off of 0.15 (dashed line) was set below the inclusion of additional reference genes was not required for normalization. Pairwise variation analysis revealed a minimum number of three reference genes for accurate normalization with the exception at 7 days post lesion, where a minimal number of four reference genes was determined.

Expression stability of seven putative RGs (*Alas1*, *Hprt*, and *Tfrc* were excluded, see above) was studied in the microdissected dentate oml after entorhinal denervation using the same approach as for the gcl, i.e., using geNorm, NormFinder and a consensus ranking analysis by RankAggreg (Table 2). In addition, expression stability of putative RGs was analyzed for the entire lesion period (all dpl) following denervation (Fig. 6a). The minimum number of RGs necessary for proper normalization was calculated by pairwise variation for all individual time points and for the entire period after denervation. As a result, three RGs were calculated to be sufficient for accurate qPCR normalization with the exception for 7 dpl, where the minimum number of four RGs was determined (Fig. 6b). NormFinder identified *Sdha* and *Actb* as the best RG pair, whereas geNorm calculated *Rpl13a*, *Sdha* and *Gapdh* as the most stable RGs suitable for accurate normalization for the entire lesion period. Summarized by RankAggreg, the consensus ranking list reads as follows (most stable to least stable): *Sdha–Ppia–Gapdh–Rpl13a–Actb–Pgk1–B2m.*

**Table 2:** Ranking of candidate reference genes according to their expression stability for the outer molecular layer after entorhinal denervation.

dpl	Ranking	NormFinder		geNorm		RankAggreg
		Stability value		M-value		Ranks
1 dpl	1	Sdha*	0.056	***Actb* + *Pgk1***	0.158	***Sdha***
	2	***Ppia***	0.066			***Pgk1***
	3	Gapdh*	0.083	***Sdha***	0.164	***Actb***
	4	***Pgk1***	0.098	***Gapdh***	0.176	***Ppia***
	5	***Actb***	0.100	***Ppia***	0.189	***Gapdh***
	6	***Rpl13a***	0.114	***Rpl13a***	0.202	***Rpl13a***
	7	***B2m***	0.152	***B2m***	0.242	***B2m***
3 dpl	1	***Ppia***	0.044	***Pgk1* + *Sdha***	0.144	***Sdha***
	2	***Sdha***	0.152			***Pgk1***
	3	***Pgk1***	0.181	***Rpl13a***	0.180	***Ppia***
	4	Rpl13a*	0.224	***Gapdh***	0.208	***Rpl13a***
	5	***Gapdh***	0.234	***Ppia***	0.232	***Gapdh***
	6	Actb*	0.236	***Actb***	0.300	***Actb***
	7	***B2m***	0.436	***B2m***	0.412	***B2m***
7 dpl	1	Actb*	0.189	***Rpl13a* + *Sdha***	0.208	***Sdha***
	2	Ppia*	0.208			***Rpl13a***
	3	***Sdha***	0.253	***Gapdh***	0.281	***Actb***
	4	***Gapdh***	0.269	***Ppia***	0.301	***Ppia***
	5	***Rpl13a***	0.277	***Actb***	0.364	***Gapdh***
	6	***Pgk1***	0.289	***Pgk1***	0.425	***Pgk1***
	7	***B2m***	0.476	***B2m***	0.523	***B2m***
14 dpl	1	Sdha*	0.044	***Actb* + *Pgk1***	0.105	***Actb***
	2	Actb*	0.092			***Sdha***
	3	***Ppia***	0.093	***Sdha***	0.153	***Pgk1***
	4	***Pgk1***	0.122	***Ppia***	0.170	***Ppia***
	5	***Rpl13a***	0.179	***Rpl13a***	0.192	***Rpl13a***
	6	***Gapdh***	0.234	***Gapdh***	0.216	***Gapdh***
	7	***B2m***	0.447	***B2m***	0.365	***B2m***
28 dpl	1	Sdha*	0.037	***Actb* + *Pgk1***	0.178	***Actb***
	2	Rpl13a*	0.077			***Pgk1***
	3	***Actb***	0.081	***Ppia***	0.196	***Sdha***
	4	***Pgk1***	0.086	***Sdha***	0.213	***Rpl13a***
	5	***Gapdh***	0.093	***Gapdh***	0.251	***Ppia***
	6	***Ppia***	0.097	***Rpl13a***	0.304	***Gapdh***
	7	***B2m***	0.248	***B2m***	0.413	***B2m***

Candidate reference genes were ranked according to their stability values by NormFinder, averaged expression by geNorm and using a consensus ranking analysis by RankAggreg for the outer molecular layer at specific time-points after entorhinal denervation. Asterisks (left column) indicate the genes selected as ‘best combination of two RGs’ by NormFinder.

### Evaluation of validated reference genes for accurate quantification of gene expression changes following entorhinal denervation

**Figure 7:**
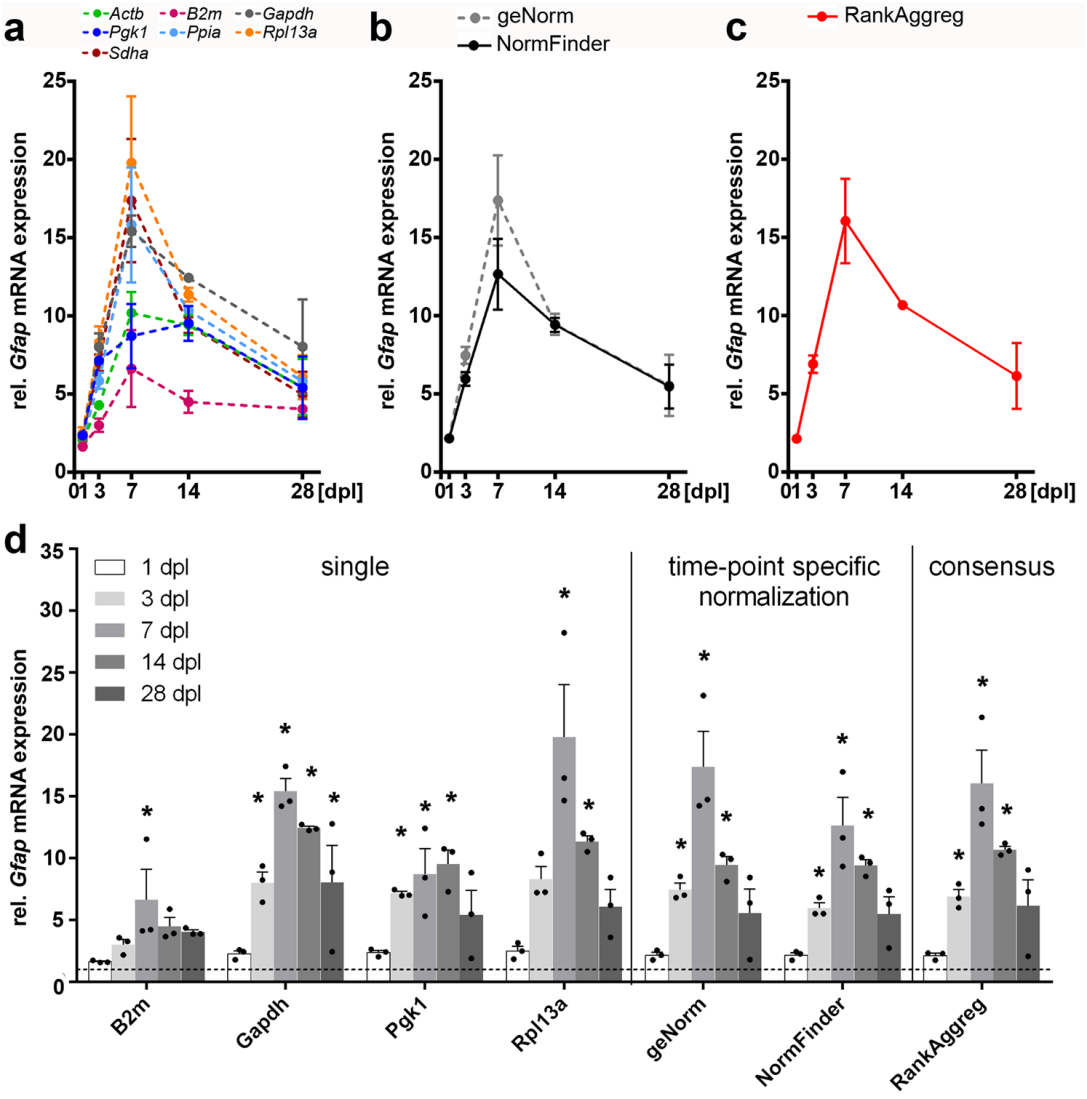
Influence of different normalization strategies for accurate quantification of *Gfap* mRNA expression levels in the dentate outer molecular layer after entorhinal denervation. (**a**, **d**) Normalization using a single reference gene resulted in different *Gfap* mRNA expression levels in the dentate outer molecular layer after entorhinal denervation. (**b**, **d**) In comparison, normalization using reference genes determined by geNorm or NormFinder for specific time-points after denervation showed a comparable time course and pattern of *Gfap* mRNA expression levels. (**c**, **d**) Results from both algorithms were combined using RankAggreg to obtain a consensus ranking for all time-points following denervation. dpl, days post lesion. Data are shown as mean ± SEM. N = 3 animals per time point. Statistics: One-way ANOVA, followed by Dunnett’s post-hoc test, with *p < 0.05.

**Figure 8:**
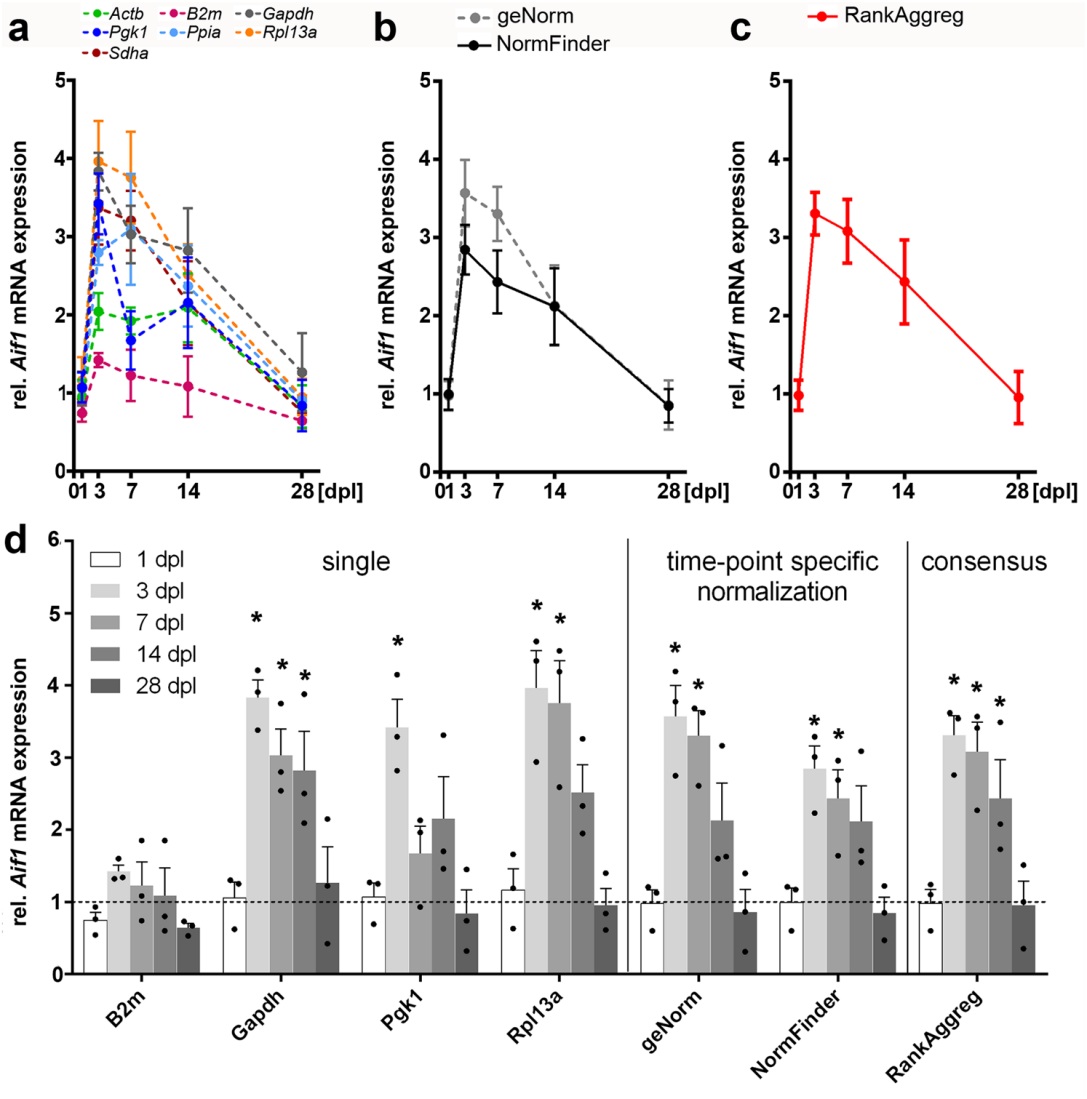
Influence of different normalization strategies for accurate quantification of *Aif1* mRNA expression levels in the dentate outer molecular layer after entorhinal denervation. (**a**, **d**) Normalization using a single reference gene resulted in different *Aif1* mRNA expression pattern in the dentate outer molecular layer after entorhinal denervation. (**b**, **d**) Normalization using reference genes determined by geNorm or NormFinder showed comparable time courses and pattern of *Aif1* mRNA expression levels. (**c**, **d**) Results from both algorithms were analyzed using RankAggreg to obtain a consensus ranking for the entire lesion period. dpl, days post lesion. Data are shown as mean ± SEM. N = 3 animals per time point. Statistics: One-way ANOVA, followed by Dunnett’s post-hoc test, with *p < 0.05.

Immunostaining for GFAP and IBA1 (see Fig. 2) shows a strong response of GFAP-positive astrocytes and IBA1-positive microglia cells in the denervated oml. To validate our normalization recommendations, we analyzed mRNA expression levels of *Gfap* and *Aif1/Iba1* in the microdissected oml at 1, 3, 7, 14 and 28 dpl. To compare the different normalization strategies, target expression levels were normalized (1) against a single candidate RG, (2) against RG indices calculated by geNorm and NormFinder for each lesion time-point, and (3) using the recommended minimal number of RGs for normalization for the entire lesion period based on the consensus ranking analysis. Normalization against a single RG resulted in high variabilities of *Gfap* mRNA expression levels in the denervated oml (Fig. 7a,d). Of note, maximal upregulation of *Gfap* mRNA was found at 7 dpl for all RGs except for *Pgk1* (Fig. 7a,d). Using an index of the most stable RGs according to NormFinder and geNorm, a very similar pattern and time course of *Gfap* mRNA expression following denervation were found for both algorithms (Fig. 7b,d). By using the best combination of RGs identified by NormFinder, a significant increase in *Gfap* mRNA expression at 3, 7 and 14 dpl (5.9-, 12.7- and 9.4-fold, respectively) was found. Likewise, using the minimal number of RGs for accurate normalization determined by geNorm, *Gfap* mRNA expression was also found to be significantly upregulated at 3, 7 and 14 dpl (7.4-, 17.37- and 9.4-fold, respectively) (Fig. 7b,d). Finally, using a consensus stability ranking taking the entire postlesion period and both software algorithms into account, an index of the three most stable RGs, i.e., *Sdha*, *Ppia* and *Gapdh,* was identified as appropriate for accurate normalization. As a result, a significant upregulation of *Gfap* mRNA at 3, 7 and 14 dpl with corresponding expression levels of 6.9-, 16.1 and 10.7-fold, respectively, were obtained (Fig. 7c,d). Likewise, the time course of *Aif1* mRNA expression was analyzed in the dentate oml after entorhinal denervation. *Aif1* mRNA was found to be upregulated at specific time points after denervation, however, to a much lower extent compared to *Gfap* mRNA expression. Normalization against a single candidate RG showed relatively variable *Aif1* mRNA expression with maximal upregulation at 3 dpl, 7 dpl or 14 dpl, depending on the RG that was applied for normalization (Fig. 8a,d). Importantly, *Aif* mRNA expression was not significantly different compared to control, if *Actb* or *B2m* were used for normalization (Fig. 8a,d). Using RGs indices of both software algorithms, a significant upregulation of *Aif1* mRNA was detected at 3 and 7 dpl (Fig. 8b,d). NormFinder identified *Rpl13a* and *Actb* at 3 dpl, and *Actb* and *Ppia* at 7 dpl as the best combination of RGs for normalization. *Aif* mRNA expression was found to be upregulated to 2.8- and 2.4-fold, respectively. Normalization with the minimal number of RGs as determined by geNorm revealed an upregulation of *Aif1* mRNA of 3.6 and 3.3-fold at 3 dpl (*Pgk1*, *Sdha* and *Rpl13a*) and 7 dpl (*Rpl13a*, *Sdha* and *Gapdh*), respectively. Finally, using a consensus stability ranking taking all time points and both software algorithms into account, an index of the most stable RGs, i.e., *Sdha*, *Ppia*, *Gapdh,* was used for normalization. Here, a significant upregulation of *Aif1* mRNA was found again at 3 dpl and 7 dpl as well as at 14 dpl with corresponding expression levels of 3.3-, 3.1- and 2.4-fold compared to control situation (Fig. 8c,d). In conclusion, a distinct time course of expression level changes for *GFAP* mRNA and for *Aif* mRNA could be identified in the dentate oml after entorhinal denervation by using indices of stable RGs for accurate qPCR normalization. Of note, maximal *Aif1* mRNA upregulation was detected at 3 dpl, while *Gfap* mRNA level was highest at 7 dpl.

To demonstrate the feasibility of our method also for neurons, we analyzed mRNA expression levels of *Map2* in the microdissected gcl at 1, 3, 7, 14 and 28 dpl. In this layer, the somata of the denervated granule cells are densely packed and microdissection is an excellent tool to harvest tissue enriched in mRNA of these neurons. Although the response of granule cells to denervation is an order of magnitude weaker than the response of glial cells and barely detectable by conventional methods such as in situ-hybridization or northern blot^45,46^, we here report changes in *Map2* mRNA in mouse granule cells following denervation (Supplementary Fig. 1). *Gapdh*, *Pgk1* and *Hprt* were used as reference genes, as determined earlier using NormFinder and geNorm. The time course based on these three reference genes suggests an upregulation of *Map2* mRNA to ~ 150% at 3 and 7 days after lesion, and a gradual decline thereafter (Supplementary Fig. 1).

## Discussion

Quantitative PCR is a highly sensitive method of choice to analyze gene expression changes, e.g., after injury of the rodent brain^16,29,37^. In recent years, it has become more and more evident that accuracy of qPCR results depends on several critical steps, in particular on the choice of appropriate internal controls, i.e., reference genes^5,47–49^. Earlier studies postulated that a single RG might be sufficient in certain experimental instances^35,50^. However, recent studies have stressed the importance to select and validate a sufficient number of RGs suitable and specific for the experimental model used^14,33,34,36,51–53^. In studies focusing on brain injury, the situation may be even more complex, since the injured brain goes through a sequence of events with time and a RG suitable for one time point after injury may not be the best RG for other time points. In the present study, we have used a well-established brain injury model, i.e., the entorhinal denervation model, as an example. Using laser microdissection, we cut out with a very high degree of specificity cell layers of the dentate gyrus affected by denervation. Further, we collected tissue at several time points after denervation, thus covering most of the time during which denervation-induced reorganization occurs. This approach demonstrated for this model that it is crucial to select RGs suitable for all time points if time points are to be compared using qPCR. Furthermore, we provided an algorithm, which others can readily adapt and transfer to other brain injury models, making qPCR data in the field of brain injury and regeneration more precise and reproducible.

### State-of-the-art software algorithms help to identify stable reference genes

Some of the most widely used RGs after entorhinal lesion are *Actb, Gapdh and Hprt*^32,54,55^. However, in those earlier studies mRNA levels were analyzed based on Cq-values relative to control without additional validation, e.g., by using advanced software algorithms like NormFinder or geNorm. These software algorithms are now state-of-the-art and changed profoundly the strategies to identify appropriate RGs^39,40^. Considering the importance of the design of qPCR experiments^5,6^, our study provides a useful algorithm to find suitable RGs for gene expression studies following brain injury or under other defined lesioning conditions. The two software algorithms used in the present study yielded similar results, with some notable differences: Because geNorm relies on pairwise comparison, it is more sensitive to co-regulated RGs. NormFinder, however, considers both the intra- and intergroup variation of RGs by calculating stability values. Thus, differences in ranking order can be explained by distinct mathematical calculations by the software tools. Therefore, we applied a consensus stability ranking analysis, which was used in previous RG validation studies^56–58^. In addition, the minimum number of RGs for accurate normalization was determined by using the recommended cut-off value of 0.15^40^. Earlier gene expression studies using brain injury models in the rodent, including the validation of suitable RGs, were mostly done in larger tissue samples, e.g., whole hippocampus^13,14,16,32,59,60^. However, RGs might be differentially expressed among distinct cell types because layers react differently to the injury. Therefore, we evaluated RGs in hippocampal layers by using the advantage of laser microdissection^29,38^.

### Comparison of candidate reference genes

Initially, the expression of up to ten selected RGs was evaluated in two DG layers, i.e., the gcl and oml, to examine for abundance and variability of RGs. Of note, a few putative RGs had to be excluded from further validation analysis in the denervated oml, since they showed insufficient amplification because of low abundance expression, which could have influenced accurate qPCR quantification. Therefore, ten candidate RGs for the gcl and seven RGs for the oml were investigated for their expression stability after entorhinal denervation. Validation of RGs was performed at five individual time points after lesion, i.e., 1, 3, 7, 14 and 28 dpl, compared to control situation. *Gapdh*, which is one of the most used RGs in qPCR, has been discussed, on the one hand, as being suitable for proper normalization^14,32,34,36,51,61^, on the other hand, it was detected as being a less stable RG in other studies^13,16,33,35,52,62^. Based on our results, *Gapdh* belongs to the suitable RGs in the gcl as well as in the oml after entorhinal denervation, if the entire time-period after injury is considered. Also, *Pgk1* was recognized as one of the most stable RGs in the gcl after entorhinal denervation. Similarly, it belongs to the most suitable RGs in the denervated oml, but except for 7 dpl, where it was found to be less stable compared to other RGs. In comparison, *Actb* was identified as least stable RG in the gcl at 1dpl and in the oml at 3 dpl, whereas at later lesion time points, it was recognized as one of the more suitable RGs in both layers. Previous studies on other brain injury models also observed an altered expression level of *Actb*, e.g., after traumatic brain injury^31,36,59^. Studying the controlled cortical impact model in mice, expression of *Actb* was increased at early time points after injury^36^. Our findings are supported by the study of Harris et al. (2009), which found that *Actb* was significantly upregulated at 2 dpl in the deafferented hippocampus, while at 7 and 15 dpl no apparent change of expression relative to control was detectable^16^. Interestingly, only *Ppia (Cyclophilin A)* demonstrated a relatively high expression stability at all lesion time points (2, 7 and 15 dpl) in this study. In our study, *Ppia* was recognized as a suitable RG in the denervated oml, too. In addition, various studies analyzed *Ppia* in other commonly used brain injury models and identified it as being one of the most stable RGs^15,33,63,64^. In contrast, *B2m* was recognized as a less stable RG in several studies^12,14,34,36,51,59,60,63^, but was postulated also as suitable RG in a few research papers^31,35,65^. In our study, *B2m* was identified as the least stable RG in the denervated oml at all time points analyzed following lesion. In contrary, *Sdha* was identified as the most stable RG in the oml throughout all time points after denervation. This is in accordance with previous brain injury studies, where *Sdha* was used as RG for accurate qPCR normalization^14,64,65^.

### Accurate measurement of denervation-induced changes in *Gfap* and *Aif1/Iba1* mRNA in the dentate gyrus using a normalization index

As proof-of-principle, we used the RGs found to be suitable for accurate qPCR normalization after denervation to analyze the expression of two mRNAs of astrocytes and microglia, i.e., *Gfap* and *Aif1/Iba1*, respectively. Both genes are upregulated in the denervated zone and reflect the strong glial response to entorhinal denervation^26,66^. Using immunofluorescence labeling for AIF1/IBA1 and GFAP, a major upregulation of these proteins in microglial cells as well as astrocytes, respectively, could also been seen at the protein level at 3 and 7 dpl in the denervated oml^29^. In previous studies, *Gfap* mRNA levels were measured in hippocampal samples using dot plot hybridization analysis^44,67^ or using laser microdissection in combination with qPCR using a single RG for qPCR normalization^24^. We now extended these earlier studies by analyzing the effect of different qPCR normalization strategies: target expression levels of *Gfap* and *Aif1* mRNA were normalized in the denervated oml (1) against single candidate RGs, (2) against RG indices calculated by geNorm or NormFinder for each specific time-point following denervation, and (3) using the minimal number of RGs calculated for accurate qPCR normalization based on a consensus stability ranking for the entire lesion period and across the software algorithms used. Normalization against a single RG demonstrated a relatively high variation of mRNA expression levels for *Gfap* and *Aif1* in the denervated oml. Significant upregulation of *Gfap* mRNA expression was found at 3, 7, 14 and 28 dpl depending on the RG that was used for normalization. Except for *Pgk1*, maximal upregulation of *Gfap* mRNA was detected at 7 dpl with expression levels varying considerably from 6.5- fold using *B2m* to 19.8- fold using *Rpl13a*. Similarly, a significant upregulation of *Aif1* mRNA expression was found either at 3, 7 or 14 dpl depending on the RG used for qPCR normalization. Maximal upregulation of *Aif1* mRNA was identified at 3 dpl using *Gapdh*, *Pgk1*, *Rpl13a* or *Sdha*. In contrast, normalization against *Actb* or *B2m* resulted in no significant expression changes of *Aif1* mRNA. Of note, *B2m* was found to be the least stable RG in the oml at all time points analyzed following denervation. Similarly, *Actb* should also not be considered at earlier time points after denervation, but might be more suitable at later time points, which was also shown in the study of Harris and colleagues (2009). Using an index of suitable RGs according to the different software approaches by NormFinder and geNorm, a significant upregulation of *Gfap* mRNA at 3, 7 and 14 dpl and for *Aif* mRNA at 3 and 7 dpl was found. Importantly, a maximal upregulation of *Aif1* mRNA was observed at 3 dpl, whereas maximal upregulation of *Gfap* mRNA upregulation was found at 7 dpl, which in both cases returned to lower levels at later time points after injury. Finally, qPCR normalization was performed using an index of the three most stable RGs, i.e., *Sdha*, *Ppia* and *Gapdh*, calculated based on a consensus stability ranking by including all lesion time-points and both software algorithms used. As a result, a significant upregulation of *Aif1* mRNA and *Gfap* mRNA was found for both transcripts at 3, 7 and 14 dpl. Again, maximal upregulation of *Aif* mRNA was observed earlier (3 dpl) compared to *Gfap* mRNA (7 dpl). These results are in line with the early response of microglia cells, followed by the later reaction of astrocytes in rodents following entorhinal lesion^42,66^. Differences in the detailed time course of *Gfap* mRNA expression might be explained by anatomical species variations between mouse and rat or by distinct lesion techniques^26^. Our results demonstrate that normalization against non-validated or relatively non-stable RGs can lead to inconsistencies in qPCR expression analysis and, consequently, might lead to misinterpretation of real biological data. In contrast, normalization against an index of validated RGs determined by advanced software algorithms yields more accurate data. In addition, the precise harvesting of tissue, e.g., by using laser microdissection, helps to identify local changes after brain injury, which is of the essence to understand the sequence of events following damage. Based on our results, a normalization index with a minimal number of suitable RGs is sufficient for accurate qPCR quantification of target genes following entorhinal denervation. For a more precise analysis, we would recommend using time-point specific reference gene sets. However, the usage of a more broader normalization index of RGs calculated by consensus stability ranking for an entire lesion period is also highly appropriate and, thus, might be used in forthcoming studies, where more lesion time-points across different subregions are analyzed.

### Changes in *Map2* mRNA in denervated granule cells are revealed using laser microdissection and qPCR in combination with a normalization index

To demonstrate the usefulness of our algorithm for the detection of neuronal genes, we also analyzed changes in *Map2* mRNA in granule cells. Microdissection of this neuronal layer is an excellent tool to harvest tissue enriched in granule cell mRNA, since granule cells are densely packed in this layer and greatly outnumber other neurons and glial cells. However, in contrast to glial cells, which react strongly to the degeneration of axon terminals and axons in the outer molecular layer, granule cells show only a very weak response. In fact, earlier studies performed in rats^25,45,46^ reported only very mild changes, which were an order of magnitude weaker than the changes found in glia and which were close or even under the threshold of detection using classical methods. Using laser microdissection and qPCR with a reference gene index established in the present study, we could, however, detect a mild upregulation of *Map2* mRNA in mouse granule cells around day 3 to 7. This demonstrates that the algorithm proposed in this study also works with neurons and most likely any other cell type and allows for the identification of an index of stable and reliable reference genes.

In conclusion, we have shown here using the well-established and thoroughly studied entorhinal cortex lesion model, that tissue from different time points after lesion harvested with laser microdissection and analyzed with qPCR yields precise, reliable, and reproducible quantitative datasets. To achieve this precision, it was of the essence to identify an index of RGs that is (i) appropriate for the model and (ii) covers the entire time course postlesion. The algorithm reported here can easily be adapted and thus transferred to any other brain injury model, making qPCR data in the field of brain injury and regeneration more precise and reproducible.

## Material and methods

### Animals

For experimental analysis, adult male mice (3 to 4-month-old C57BL/6 J, Janvier) were used. Mice could survive 1, 3, 7, 14 or 28 days after entorhinal denervation. Experiments were carefully designed to examine control and lesioned animals of similar age.

### Ethical approval

Animal care and experimental procedures were performed in agreement with the German law on the use of laboratory animals (animal welfare act; TierSchG) and approved by Regierungspräsidium Darmstadt (Hessen, Germany). The animal experiments described in this study were conducted in accordance with ARRIVE guidelines.

### Entorhinal denervation and control of lesion quality

Unilateral transection of the perforant path was performed using a wire knife (David Kopf Instruments, USA) as described previously^29^. Correct placement of the wire knife cut was verified on horizontal brain sections (25 µm, Nissl staining) containing the lesion site and parts of the temporal dentate gyrus (DG). In addition, entorhinal denervation was verified on frontal hippocampal sections (25 µm) using Fluoro-Jade C (HistoChem Inc.) at early time points post lesion to monitor the appearance of degeneration products^29,30,68^ and counterstained with Hoechst 33242 (Invitrogen).

### Immunofluorescence labeling

Mice were deeply anesthetized with an overdose of pentobarbital (300 mg/kg body weight) and transcardially perfused with 0.9% sodium chloride (NaCl) followed by 4% paraformaldehyde (PFA) in phosphate-buffered saline (pH 7.4). Brains were removed, post-fixed for 24 h in 4% PFA and sectioned in the coronal plane (40 µm) using a vibratome (VT1000 S, Leica Microsystems). Free-floating sections were incubated in a blocking buffer containing 0.5% Triton X-100 and 5% bovine serum albumin (BSA) in 0.05 M Tris-buffered saline (TBS) for 30 min at room temperature followed by incubation in the primary antibody (diluted in 0.1% Triton X-100 and 1% BSA in 0.05 M TBS) overnight at 4 °C. The following primary antibodies were used: rabbit anti-GFAP (immunogen: GFAP isolated from cow spinal cord; Z0334, Dako), mouse anti-NeuN (A60, immunogen: purified cell nuclei from mouse brain; MAB377, Chemicon) and anti-AIF1/IBA1 (1:100, Abcam, UK). After several washes, sections were incubated with the appropriate secondary Alexa-conjugated antibodies (1:2000, Invitrogen, USA) for several hours at room temperature and finally mounted in DAKO Fluorescent Mounting Medium (Dako).

### Digital illustrations

Figures were prepared digitally using commercially available graphics software (Photoshop Adobe Inc.). Fluorescent images were acquired using a digital camera (Digital Sight DS-5Mc, Nikon, Germany) or confocal microscopy (Eclipse C1 Plus, Nikon). Single fluorescent images of the same section were digitally superimposed. The contrast, brightness and sharpness of images were adjusted as needed for each section. No additional image alteration was performed.

### Laser microdissection

For layer-specific analysis of the granule cell layer (gcl) and outer molecular layer (oml) of the DG, prepared brains were embedded in tissue freezing medium and immediately flash-frozen in − 70 °C isopentane cooled by dry ice for 2 min. Until further processing, brains were transferred and stored at − 80 °C. For laser microdissection, 16 µm thin brain sections were cut using a cryostat (Leica Biosystems) and mounted on polyethylene naphthalene (PEN) membrane slides (Leica Microsystems). Sections were dried shortly at room temperature (RT), fixed in − 20 °C cold 75% and 100% ethanol (AppliChem) and stored at − 80 °C until further processing. Before laser microdissection, sections were thawed and stained quickly with 1% cresyl violet staining solution (Sigma-Aldrich) at RT and briefly dehydrated in 75% and 100% ethanol. Using a Leica LMD6500 system (Leica Microsystems), defined tissue portions of the gcl and oml of the DG from the same brain sections were collected separately in 50 µl lysis buffer (RNeasy Plus Micro Kit; Qiagen) with ß-mercaptoethanol. Tubes were refilled to 350 µl with lysis buffer, vortexed for 30 s and transferred immediately to − 80 °C until further processing. Total RNA was isolated using the RNeasy Plus Micro Kit (Qiagen) according to the manufacturer’s recommendations. RNA integrity was assessed using the Agilent 2100 Bioanalyzer system and Agilent RNA 6000 Pico Kit (Agilent Technologies). Only high RNA quality (RIN values > 8) samples were used for further processing.

### Quantitative polymerase chain reaction

Total RNA from microdissected DG regions was reverse transcribed using HighCapacity cDNA Reverse Transcription Reagents Kit (Applied Biosystems) following the manufacturer’s recommendations. Quantitative PCR (qPCR) was performed using the StepOnePlus Real-Time PCR System (Applied Biosystems). qPCR conditions were carried out using EXPRESS SYBR GreenER qPCR SuperMix with Premixed Rox (Invitrogen) and a final primer concentration of 500 nM. Primers were designed to be intron-spanning to exclude amplification of genomic DNA. Ten potential reference genes (RGs) were selected for analysis: actin, beta (*Actb*), aminolevulinic acid synthase 1 (*Alas1*), beta-2 microglobulin (*B2m*), glyceraldehyd-3-phosphate dehydrogenase (*Gapdh*), hypoxanthine guanine phosphoribosyl transferase (*Hprt*), phosphoglycerate kinase I (*Pgk1*), peptidyl propyl isomerase A (*Ppia*), ribosomal protein L13A (*Rpl13a*), succinate dehydrogenase complex subunit A (*Sdha*) and transferrin receptor (*Tfrc*) (Table 3). Amplicon specificity was confirmed by melting curve analysis. In addition, PCR products were verified for product specificity and amplicon size with the Agilent 2100 Bioanalyzer system using Agilent DNA 1000 Chips (Agilent Technologies). Primer efficiencies and quantification cycle (Cq) values were calculated using LinRegPCR^69,70^.

**Table 3:** Details of qPCR assays for candidate reference genes and target genes.

Symbol	Gene name	Accession number	Primer sequence forward and reverse	Amplicon size [bp]	Efficiency (LinRegPCR)
***Actb***	actin, beta	NM_007393.5	GAAGATCAAGATCATTGCTCCT	84	1.96
			TGGAAGGTGGACAGTGAG		
***Alas1***	aminolevulinic acid synthase 1	NM_020559.2	CGATGCCCATTCTTATCC	75	1.95
			TTGAGCATAGAACAACAGAG		
***B2m***	beta-2 microglobulin	NM_009735.3	CCTCTGTACTTCTCATTACTTG	92	1.97
			GCCTCTTTGCTTTACCAA		
***Gapdh***	hypoxanthine guanine phosphoribosyl transferase	NM_008084.3	ACAATGAATACGGCTACAG	78	1.94
			GGTCCAGGGTTTCTTACT		
***Hprt***	glyceraldehyde-3-phosphate dehydrogenase	NM_013556.2	GTGATTAGCGATGATGAAC	117	1.95
			TTCAGTCCTGTCCATAATC		
***Pgk1***	phosphoglycerate kinase 1	NM_008828.3	CGTGATGAGGGTGGACTT	79	1.98
			TGGAACAGCAGCCTTGAT		
***Ppia***	peptidylprolyl isomerase A	NM_008907.2	CAAGACTGAATGGCTGGAT	75	1.95
			ATGGCTTCCACAATGTTCA		
***Rpl13a***	ribosomal protein L13A	NM_009438.5	TCCACCCTATGACAAGAA	85	1.95
			GTAAGCAAACTTTCTGGTAG		
***Sdha***	succinate dehydrogenase complex, subunit A, flavoprotein (Fp)	NM_023281.1	CAAGACTGGCAAGGTTAC	101	1.95
			ATCAGTAGGAGCGGATAG		
***Tfrc***	transferrin receptor	NM_011638.4	CCGACAATAACATGAAGG	127	1.94
			TTACAATAGCCCAGGTAG		
***Gfap***	glial fibrillary acidic protein	NM_010277.3 NM_001131020.1	AACCTGGCTGCGTATAGA	125	2
			CGAACTTCCTCCTCATAGAT		
***Aif1***	allograft inflammatory factor 1	NM_019467	ATCAACAAGCAATTCCTC	116	1.97
			ATATCTCCATTTCCATTCAG		

### Data analysis

To evaluate the gene expression stability of candidate RGs, two different software algorithms, i.e., NormFinder^39^ and geNorm^40^, were used according to the developer’s manuals. NormFinder calculates a stability value for each gene independently and matches combinations of gene pairs to compensate for variability, whereas geNorm calculates an average expression stability value (*M*), which is the average pairwise variation of a single gene to all other putative RGs and is the result of a stepwise exclusion of the least stable gene within the panel of RGs. In addition, to reach an unbiased consensus for both software algorithms, a comprehensive ranking analysis was performed using RankAggreg^41^. Minimum number of RGs for accurate normalization was calculated based on optimal gene rank lists by pairwise variation between two sequential normalization factors containing an increasing number of genes^40^. Based on this study, 0.15 was set as a cut-off, below the inclusion of an additional RG was not required for proper normalization. Statistical analysis of qPCR data was performed using GraphPad Prism 6 software. For one-way ANOVA, multiple comparisons between individual time-points and control were used in combination with Dunnett’s multiple comparisons test. *P*-values < 0.05 were considered statistically significant.

## Supplementary Information

Supplementary Information 1.

Supplementary Information 2.

## Data Availability

The datasets used and/or analyzed during the current study available from the corresponding author on reasonable request.
