# Supplementary material for: Precise measurement of gene expression changes in mouse brain areas denervated by injury
Source: Sci Rep. 2022 Dec 29;12:22530. doi: 10.1038/s41598-022-26228-5 (PMC9800364; doi:10.1038/s41598-022-26228-5)
Supplement: Supplementary file 1 — Supplementary Information 1. [file 41598_2022_26228_MOESM1_ESM.docx]

**Supplementary Figure legend**

**Supplementary Figure 1. Quantification of neuronal *Map2* mRNA expression levels in the granule cell layer after entorhinal denervation**

(a) Normalization using a single reference gene resulted in different *Map2* mRNA expression pattern in the granule cell layer after entorhinal denervation. (b, c) Normalization using reference genes determined by geNorm or NormFinder and combined in a consensus ranking using RankAggreg over all time points obtained upregulation of *Map2* mRNA expression level after denervation at 3 and 7 dpl. Quantification was performed with TaqMan Gene Expression assays (*Gapdh*, Mm99999915_g1; *Pgk1*, Mm00435617_m1; *Hprt1*, Mm01318743_m1 and *Map2*, Mm00485231_m1) following the manufacturer’s recommendations. dpl, days post lesion. Data are shown as mean ± SEM. N = 5 animals for control group and N=2-3 per lesion time point. Statistics: One-way ANOVA, followed by Dunnett’s post-hoc test, with * p <0.05.
