# Supplementary figures and images for: Precise measurement of gene expression changes in mouse brain areas denervated by injury
Source: Sci Rep. 2022 Dec 29;12:22530. doi: 10.1038/s41598-022-26228-5 (PMC9800364; doi:10.1038/s41598-022-26228-5)

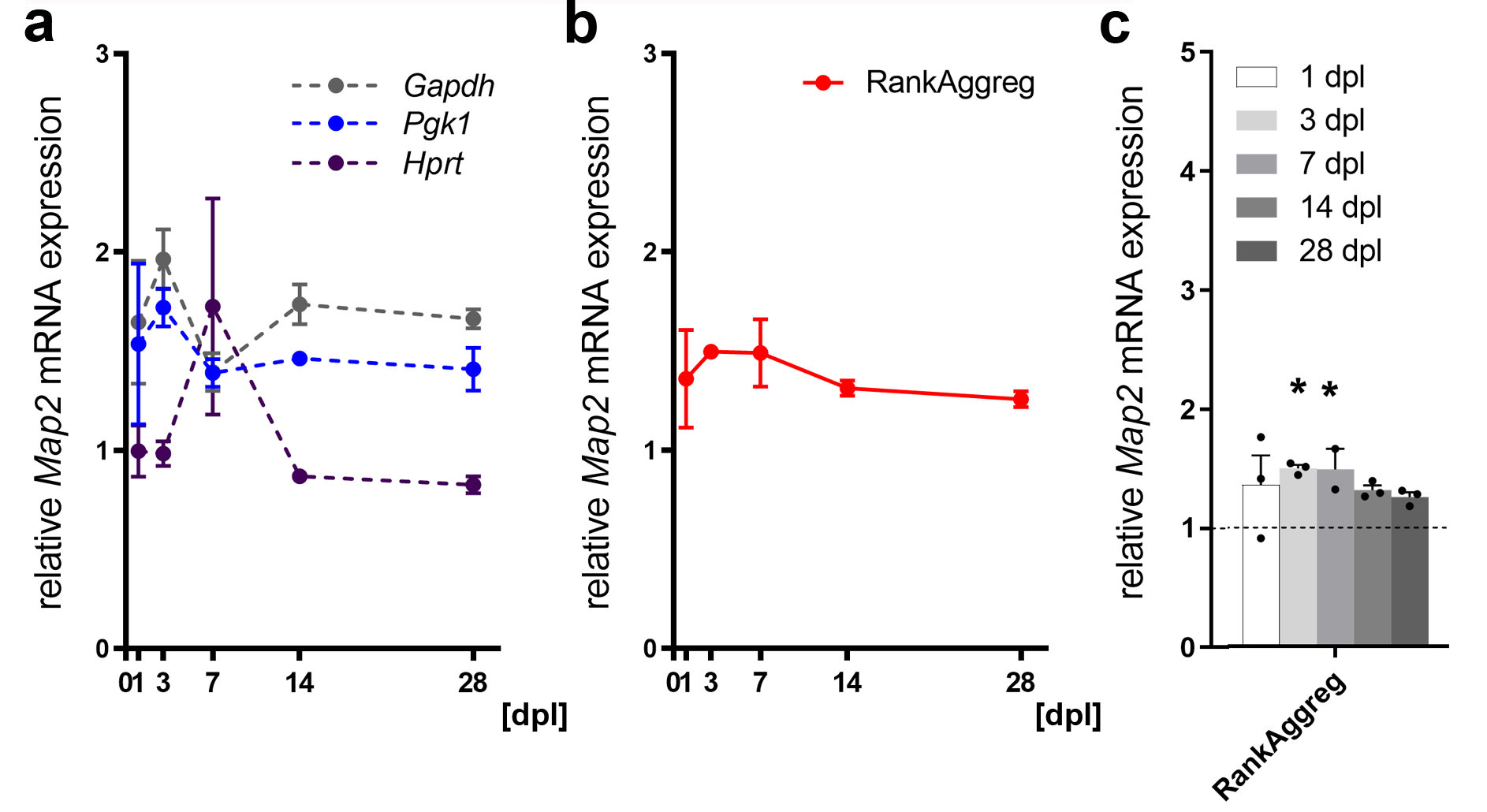

Supplement: Supplementary file 2 — Supplementary Information 2. [file 41598_2022_26228_MOESM2_ESM.tiff]
